# Structures of Coxsackievirus, Rhinovirus, and Poliovirus Polymerase Elongation Complexes Solved by Engineering RNA Mediated Crystal Contacts

**DOI:** 10.1371/journal.pone.0060272

**Published:** 2013-05-08

**Authors:** Peng Gong, Matthew G. Kortus, Jay C. Nix, Ralph E. Davis, Olve B. Peersen

**Affiliations:** 1 Department of Biochemistry and Molecular Biology, Colorado State University, Fort Collins, Colorado, United States of America; 2 Molecular Biology Consortium, Advanced Light Source, Lawrence Berkeley National Laboratory, Berkeley, California, United States of America; 3 Cocrystal Discovery Inc., Mountain View, California, United States of America; Centro de Biología Molecular Severo Ochoa (CSIC-UAM), Spain

## Abstract

RNA-dependent RNA polymerases play a vital role in the growth of RNA viruses where they are responsible for genome replication, but do so with rather low fidelity that allows for the rapid adaptation to different host cell environments. These polymerases are also a target for antiviral drug development. However, both drug discovery efforts and our understanding of fidelity determinants have been hampered by a lack of detailed structural information about functional polymerase-RNA complexes and the structural changes that take place during the elongation cycle. Many of the molecular details associated with nucleotide selection and catalysis were revealed in our recent structure of the poliovirus polymerase-RNA complex solved by first purifying and then crystallizing stalled elongation complexes. In the work presented here we extend that basic methodology to determine nine new structures of poliovirus, coxsackievirus, and rhinovirus elongation complexes at 2.2–2.9 Å resolution. The structures highlight conserved features of picornaviral polymerases and the interactions they make with the template and product RNA strands, including a tight grip on eight basepairs of the nascent duplex, a fully pre-positioned templating nucleotide, and a conserved binding pocket for the +2 position template strand base. At the active site we see a pre-bound magnesium ion and there is conservation of a non-standard backbone conformation of the template strand in an interaction that may aid in triggering RNA translocation via contact with the conserved polymerase motif B. Moreover, by engineering plasticity into RNA-RNA contacts, we obtain crystal forms that are capable of multiple rounds of in-crystal catalysis and RNA translocation. Together, the data demonstrate that engineering flexible RNA contacts to promote crystal lattice formation is a versatile platform that can be used to solve the structures of viral RdRP elongation complexes and their catalytic cycle intermediates.

## Introduction

The replication of positive-strand RNA viruses relies on virally encoded RNA-dependent RNA polymerases (RdRPs) that are capable of rapid and processive synthesis of the 7–14 kilobase viral genomes. However, they do so with the fairly low fidelity of one error in every 10^4^–10^5^ bases synthesized, and consequently these viruses have been described as “quasi-species” where individual members of the virus population have some distribution of point mutations relative to a consensus sequence [1]. The existence of this genetically diverse population is essential for efficient virus growth because it allows the virus to rapidly adapt to different hosts, cell types, and intracellular environments in the course of infection. Limiting the quasi-species variation of poliovirus with a high fidelity polymerase variant prevents the spread of the virus within a mouse model [2]–[4]. Reducing fidelity is also deleterious for virus growth, as shown by recent coxsackievirus studies where low fidelity polymerase variants resulted in viruses that replicate well in tissue culture, but then rapidly extinguish and fail to establish persistent infections in animal models [5]. The quasi-species population is also essential for the growth and fitness of zoonotic arboviruses, such as chikungunya virus, that must maintain genetic diversity while replicating in both avian and mosquito environments [6].

The molecular basis for nucleotide selection and catalysis by these viral RdRPs was demonstrated by a series of crystal structures of the poliovirus polymerase (3D^pol^) elongation complex [7]. Starting with the same initial crystal form and soaking the crystals with NTPs and deoxy-NTPs, we captured a series of distinct polymerase conformations that showed the conformational changes responsible for nucleotide recognition and subsequent closure of the active site for catalysis. These data led to a model for RdRP catalytic cycle comprised of six core structural states that likely represent the main intermediates implied by kinetic studies of RdRP catalysis, although this correspondence has not been explicitly shown [7]. The relative simplicity of the interactions and conformational changes associated with this catalytic cycle model are likely major contributors to the low fidelity of the viral RdRPs, and these enzymes do not have a post-catalysis proofreading mechanism.

The catalytic cycle model starts with State **1** that is a paused replicative polymerase with two unusual features that are unique to the viral RdRP enzymes; first, the templating base is already stacked on the upstream RNA duplex (i.e. the template-product duplex) and fully poised for NTP binding to the active site, and second the active site itself is not structured for catalysis because the motif A is not hydrogen bonded to the motif C, disrupting the palm domain RRM-fold common to polymerase active sites. NTP then enters the polymerase complex, where it base pairs with the templating base and base stacks on the priming base, creating a State **2** structure wherein the active site RRM fold is still not fully formed. Next, State **3** is formed by a movement of motif A in the palm domain that fully structures the RRM-fold, brings in the magnesium ions and coordinating carboxylic acids needed for catalysis, and slightly repositions the bound nucleotide for catalysis. The result is a *closed* active site that has been captured at the pre-catalysis State **3** in a norovirus polymerase-RNA-CTP complex obtained by cocrystallization [8] and at a post-catalysis State **4** in the poliovirus elongation complex after CTP soaking. Outside of the changes in covalent bonding, the structural arrangement of the polymerase-NTP-metal complex atoms at the active site appears to be essentially unchanged between a pre-catalysis state **3** and the post-catalysis state **4**. This is followed by state **5** where the active site has returned to the *open* conformation because motifs A and C of the RRM fold are no longer hydrogen bonded to each other, but the newly incorporated nucleotide and pyrophosphate product are still in the active site. The final step in the model is then State **6** that accounts for translocation of the RNA by one base pair to position the next templating nucleotide in the active site. Thus far there are no structures of translocation intermediates and it is not yet known if translocation in these RdRPs is a power-stroke type movement to rapidly translocate the RNA in a single step or a series of Brownian ratchet type sub-steps that more gradually move the RNA into its final translocated register.

Based on RdRP structure comparisons, this palm-domain based active site rearrangement appears to be unique to positive-strand RNA virus polymerases: The *open* conformation active site has been observed in all the RdRP alone and several RdRP-RNA and RdRP-RNA-NTP complex structures solved thus far, while the *closed* conformation has only been seen in the presence of RNA and cognate NTP bound at the active site, where it was trapped in the pre-catalytic state **3** in norovirus polymerase [8] and in the post-catalytic state **4** in the poliovirus elongation complex [7]. Active site closure appears to be largely driven by interactions between the enzyme and the ribose hydroxyl groups of the incoming NTP that stabilize the subtle restructuring of the palm domain that results in the formation of a functional active site. Consistent with this, the specific role of ribose 2′-hydroxyl recognition and the palm domain closure mechanism was recently addressed in a mutational study of coxsackievirus polymerase that showed a good correlation between *in vivo* virus mutation rates obtained by sequencing progeny virus genomes and the *in vitro* ability of the viral polymerases to discrimination between CTP and 2′-deoxy-CTP [5]. Over the past few years there have also been multiple reports of seemingly isolated mutations within both the palm and fingers domains that alter picornaviral polymerase fidelity [5], [9]–[14], and together these data suggest nucleotide selection and incorporation is a distributed property that utilizes many aspects of polymerase structure. These findings are also in concordance with molecular dynamics [15], [16] and NMR studies [17] showing that there is long range modulation of protein dynamics within 3D^pol^ such that residues located far from the active site can have significant effects on catalysis.

**Figure 1:**
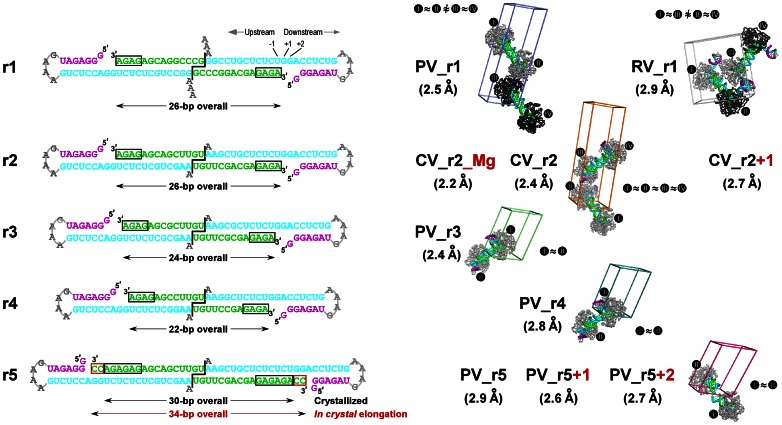
Picornaviral elongation complexes crystallized via engineered RNA-RNA contacts. **Left:** Sequences of the five RNA constructs (r1–r5) used to generate the EC crystals cartooned as coaxially stacked pairs that reflect the dumbbell-type packing seen in all the crystals. To generate the ECs, the product/primer strand was extended by 4–6 nucleotides (black boxes) via the addition of GTP and ATP prior to ion exchange purification followed by crystallization. Template, product/primer, and non-template strands are shown in cyan, green, and purple, respectively, and the 3′ dangling nucleotide(s) of the template strand and the downstream tetra-loop are shown in grey. For r2 and r5 constructs, an additional two base in-crystal extension occurred upon nucleotide soaking (red boxed). The r2–r5 constructs have two G:U base pairs at the RNA-RNA junction to increase flexibility relative to the original r1 RNA with two G:C base pairs and the constructs all have the same sequence for the downstream stem-loop element. **Right:** Space group P1 unit cells showing the EC packing arrangements of the new structures compared to the original PV_r1 poliovirus EC structure (PDB code 3OL6). The structures are named with a two-letter code to represent the virus species (PV: poliovirus; CV: coxsackievirus B3; RV: human rhinovirus 16) followed by r1–r5 to indicates the RNA construct and +N to denote any in-crystal elongation. Each EC is labeled with a roman numeral and complexes between which non-crystallographic symmetry (NCS) was used during refinement are indicated by ≈ signs and identical polymerase coloring (light grey vs. dark grey). Note that the NCS pairs in the RV_r1 structure are in different dumbbell pairs while those of the other structures are within each dumbbell pair. All EC comparisons in this paper are based on the six structures pictured here. The not pictured structures (CV_r2_Mg, CV_r2+1, PV_r5, and PV_r5+1) are only used when discussing the magnesium binding site and in-crystal elongation.

The original crystallization of the poliovirus EC appeared to be in large part driven by the coaxial stacking of the upstream RNA duplexes emerging from two different complexes. This resulted in a “dumbbell” shaped minimal crystallization unit composed of a bipartite central RNA helix segment with polymerase molecules facing away from each other at each end of the RNA helix (Figure 1). The ECs were enzymatically active in the context of the crystal lattice and would incorporate a single nucleotide upon soaking with CTP, but they were not capable of translocating the RNA after nucleotide addition. Instead, the data revealed the state **5** structure wherein the post-catalysis active site had returned to the open conformation in the absence of translocation. This is intriguing because these two events, opening the active site and translocating the nucleic acid, are usually tightly coupled in replicative polymerases, such at T7 RNA polymerase and Pol I polymerase, via interactions involving swinging motions of a major α-helix motif within the fingers domain [18]–[20]. However, the viral RdRPs do not contain such a helical structural element and their fingers domains are tethered to their thumb domains and thus unable to swing, suggesting they carry out translocation by a different mechanism. In the case the poliovirus EC structure, the lack of translocation was attributed to the coaxial stacking of the product helices from two different complexes that likely restricting the relative movement of the RNA and polymerase.

To examine this in further detail and to validate the polymerase–RNA interactions seen in the initial poliovirus EC structures, we here present nine new coxsackievirus, rhinovirus, and poliovirus elongation complex structures at resolutions ranging from 2.1 to 2.9 Å. The crystals were obtained by expanding on our initial EC purification methodology to systematically vary the length of the upstream RNA and the flexibility of the RNA-RNA junction during crystallization screening. The RNA-RNA contacts of the resulting structures exhibit a wide range of geometries and some of them form lattices that allow for multiple rounds of in-crystal catalysis and translocation while retaining diffraction. A comparison of the RNA structure in the resulting picornaviral polymerase elongation complexes shows a non-canonical RNA backbone conformation in the active site itself and that the conformation of the upstream duplex is essentially identical for 7–8 base pairs that make intimate contact with the picornaviral polymerases, but beyond this point the RNA adopts a wide range of crystal packing arrangements. Similarly, a comparison of the three different polymerases in these ECs highlight conserved interactions with the downstream RNA template, a consistent opening of the RNA exit channel to accommodate the duplex, and an *open* conformation active site in the absence of added NTP.

## Materials and Methods

Poliovirus (PV) and coxsackievirus B3 (CV) polymerases (3D^pol^) with C-terminal GSSS-His_6_ tags were produced in *E. coli* using a ubiquitin fusion construct that is post-translationally cleaved in the expression strain to generate 3D^pol^ molecules with the required glycine as the first residue of the protein [21]. They were then purified using microfluidizer based lysis followed by polyethyleneiminie (PEI) precipitation and nickel affinity, anion exchange, and size exclusion chomatography as previously described [22], [23].

Human Rhinovirus 16 polymerase (RV) was expressed from an *E. coli* optimized gene synthesized by Blue Heron Biotech (www.blueheronbiotech.com) that was cloned into the NcoI and XhoI sites of the arabinose-inducible pBADb vector from Invitrogen (www.invitrogen.com), verified by sequencing, and then transformed into TOP10 cells. Optimal arabinose levels, induction temperature (15–18°C), and growth time (15–18 hours) were determined empirically. Protein production scale cultures were done in 4-liter flasks with 800 ml of terrific broth at 250-rpm agitation. Cultures were induced with 0.2% arabinose at an OD_600_ of 1.5 and grown for 15–18 hours at 15°C. Cells were spun down and resuspended in 3 volumes of 50 mM HEPES pH 7.4, 200 mM NaCl, 5% glycerol, 1 mM EDTA, 5 mM DTT and flash-frozen in liquid nitrogen. Upon thawing, 0.1 mg lysozyme per milliliter of slurry was added and incubated on ice for 45 minutes. Lysate was sonicated briefly to decrease viscosity and a 10% solution of PEI (pH 7.8) was stirred in over 15 minutes to a final concentration of 0.4%. Cell debris and nucleic acids were pelleted by centrifugation at 15,000×g for 30 minutes. PEI and lysozyme were removed by passing clarified extract through fast flow SP sepharose. The flow-through fraction was diluted with three volumes of Buffer A (20 mM HEPES pH 7.4, 5% glycerol, 1 mM EDTA, 1 mM DTT), loaded onto a fresh fast flow SP sepharose column, washed with five volumes of BufferA+50 mM NaCl, and subsequently eluted with BufferA+200 mM NaCl. Eluted RV16 polymerase was diluted with an equal volume BufferA and loaded onto a HiTrap Heparin column for buffer change. The heparin column was washed with BufferB-50as (20 mM Tris pH 8.0, 50 mM ammonium sulfate, 5% glycerol, 1 mM EDTA, and 1 mM DTT). RV 3D^pol^ was eluted in a linear gradient of increasing ammonium sulfate, peak fractions were pooled and diluted to 50 mM ammonium sulfate in BufferB-0, loaded onto a HiTrap Q column, and eluted in a linear gradient of increasing ammonium sulfate. Pooled fractions were concentrated to 10 mg/ml and loaded onto a Superdex 75 column pre-equilibrated with BufferB-200as. Eluted monomeric 3D^pol^ was concentrated to 20–25 mg/ml and flash frozen in aliquots.

ECs for crystallization were generated and purified using minor variations of the methods previously described for poliovirus polymerase [7]. Briefly, 36 µM PV 3D^pol^, 30 µM pre-annealed primer-template RNA, and 300–450 µM each ATP and GTP were incubated at room temperature for 20–45 minutes to form stable ECs. CV 3D^pol^ is less soluble than its PV counterpart and assembly reactions where therefore done with 20 µM CV 3D^pol^ and 15 µM RNA in 50 mM HEPES, pH 6.5, 75 mM NaCl, 5 mM MgCl_2_, 2 mM TCEP, and 450 µM each ATP and GTP. Optimized conditions for the RV EC involved the addition of 10% glycerol to increase solubility of the polymerase under low salt conditions and substitution of Tris pH 7.8 and KCl for HEPES pH 6.5 and NaCl, respectively, in both the primer/template annealing reaction and EC assembly buffers. RV EC assembly was done with 20 µM 3D^pol^ and 10 µM RNA in a total volume of 9 ml and after incorporation of the first four nucleotides (GAGA), the pH of the reaction was adjusted to 6.5 by adding 1 M MES pH 6.0 as needed (≈75 mM final concentration) before the following purification steps.

The assembled EC’s were purified by ion exchange chromatography on a MonoQ column (GE Healthcare), buffer exchanged into 20 mM HEPES, pH 6.5, 100 mM NaCl, 2 mM MgCl_2_, 2 mM TCEP reducing agent, and then concentrated in Amicon Ultra-4 centrifugal filters to ≈10 mg/ml for PV and ≈8 mg/ml for CV and RV complexes. Crystallization screens were carried out using the Qiagen Cryos Suite at 16°C. If needed, promising initial conditions were optimized, resulting in final crystallization complexes for the various constructs as outlined below. Unless otherwise noted, NTP or deoxy-NTP soaking experiments were done under the final cryo stabilizer conditions using 5 mM NTP or deoxy-NTP and 10 mM MgCl_2_ for 15–20 hours.

Poliovirus PV_r3 and PV_r4 crystals grew in 0.16 M MgCl_2_, 0.08 M Tris-HCl 8.5, 24% (w/v) PEG 4000, 20% (v/v) glycerol and were directly frozen for data collection. The PV_r5 crystals were obtained using 4.8%(v/v) isopropanol, 0.096 M tacsimate (Hampton Research), pH 8.5–9.0, 1.92–1.95 M ammonium sulfate, and 10–11%(v/v) glycerol and then gradually exchanged into a cryo stabilizer solution containing 4.8%(v/v) isopropanol, 0.096 M tacsimate, pH 8.5–9.0, 1.95 M ammonium sulfate and 19–27%(v/v) xylitol prior to freezing. The coxsackievirus CV_r2 crystals grew from 0.17 M sodium acetate, 0.085 M Tris pH 8.5, 25.5% PEG 4000, and 15% glycerol and were directly frozen. Coxsackievirus CV_r2_Mg crystals grew from 0.21 M magnesium acetate, 0.08 M sodium cacodylate pH 6.5, 20% glycerol, and 14% PEG 8000 and were directly frozen. Finally, the rhinovirus RV_r1 crystals grew in 2–3 days from 0.16 M ammonium sulfate, 0.08 M sodium acetate, pH 4.6, 20% (w/v) PEG 4000, and 20% (v/v) glycerol and were directly frozen. Note that the PV polymerase used to assemble the PV_r5 complex is with a C290M mutation that produced higher quality crystals than the wild type polymerase.

Diffraction data were obtained using beamline 4.2.2 at the Advanced Light Source (Berkeley, CA). 180–185° of oscillation data were typically collected at 0.5–1° steps and the data were integrated, merged, and scaled using d*Trek [24] with the resulting statistics being listed in Table 1. Molecular replacement was done with the original PV EC structure (PDB code 3OL6) as a search model, structure refinement was done using Phenix [25], model building was done using Coot [26], and simulated annealing composite omit maps were generated using CNS [27]. All the datasets were reduced in the P1 space group, although the CV and RV datasets appear to be very close to C2 or related higher symmetry space groups. This is similar to what was observed with the original PV structures where the use of P1 revealed subtle differences in crystal packing environments that in turn affected fingers domain movements involved in NTP positioning and incorporation efficiency. To preserve the ability to detect any such subtle changes in these new crystal forms we therefore chose to reduce the data in P1 and apply non-crystallographic symmetry to related polymerase and RNA molecules during refinement as warranted. The PDB file chain assignments are consistently named with sequential letters for the protein, template, product, and non-template RNA strands within each EC (i.e. EC_I_ = A–D, EC_II_ = E–H, …). The residue numbering used for the nucleic acid in all the complexes is always indexed such that the priming −1 position base pair is composed of nucleotide 601 on the template strand, 701 on the product strand, and 702 for any incoming nucleotide in the catalytic site, regardless of nucleotide sequence, pre- or post-catalysis state, or in-crystal translocation status.

**Table 1:** Crystallographic Data and Refinement Statistics for Picornaviral EC Structures.

Complex	PV_r3	PV_r4	PV_r5	PV_r5+1	PV_r5+2	CV_r2	CV_r2+1	CV_r2_Mg	RV_r1
PDB Code	4K4S	4K4T	4K4U	4K4V	4K4W	4K4X	4K4Y	4K4Z	4K50
Space group	P1	P1	P1	P1	P1	P1	P1	P1	P1
Unit Cell									
a,b,c (Å)	58.8, 58.9, 97.6	59.4, 59.3, 97.5	63.7, 63.9, 105.1	63.5, 63.5, 102.0	64.5, 64.4, 102.3	61.0, 61.0, 195.1	60.4, 60.4, 194.0	60.6, 60.6, 194.7	53.8, 113.9, 122.6
α,β,γ (°)	77.9, 77.8, 80.0	78.1, 78.1, 78.9	101.1, 106.2, 103.0	73.5,73.4, 73.6	73.7, 73.6, 72.2	90.0, 90.0, 78.4	89.9, 90.0, 79.8	90.0, 89.9, 78.7	90.0, 90.5, 90.0
Wavelength (Å)	1.00	1.00	1.00	1.00	1.00	1.00	1.00	1.00	1.00
**Data Reduction**									
Resolution (Å)^1^	39.9–2.40(2.49–2.40)	47.0–2.75(2.85–2.75)	45.7–2.85(2.95–2.85)	47.5–2.63(2.72–2.63)	42.4–2.69(2.79–2.69)	48.8–2.37(2.45–2.37)	43.8–2.72(2.82–2.72)	38.0–2.17(2.25–2.17)	49.1–2.93(3.03–2.93)
Unique reflections	47,355	31,399	34,187	41,557	39,877	109,647	70,840	140,661	61,473
Completeness (%)^1^	97.7 (97.5)	96.1 (95.6)	98.3 (98.1)	98.0 (97.7)	97.9 (98.0)	98.0 (96.6)	97.8 (97.5)	97.8 (97.1)	98.4 (98.3)
R_merge_ 1	0.066 (0.37)	0.069 (0.33)	0.050 (0.32)	0.042 (0.32)	0.050 (0.33)	0.064 (0.30)	0.097 (0.30)	0.055 (0.34)	0.108 (0.31)
R_meas_ 1	0.093 (0.52)	0.097 (0.47)	0.070 (0.44)	0.059 (0.45)	0.070 (0.46)	0.090 (0.42)	0.137 (0.42)	0.078 (0.47)	0.153 (0.44)
Redundancy^1^	1.91 (1.92)	1.91 (1.91)	2.01 (2.02)	1.99 (2.00)	1.99 (1.98)	1.99 (1.94)	1.92 (1.92)	1.95 (1.94)	1.98 (1.98)
I/Sigma^1^	6.2 (1.5)	6.8 (1.8)	7.9 (1.7)	8.4 (1.5)	8.4 (1.5)	6.8 (1.5)	5.0 (1.6)	7.7 (1.5)	5.1 (1.6)
Mosaicity (°)	1.09	1.35	0.94	0.81	0.75	0.34	0.93	0.32	0.50
**Structure Refinement**									
R_factor_ (%)	19.8	20.2	21.9	23.3	21.3	20.4	19.9	20.4	19.1
R_free_ (5% of data) (%)	24.5	26.2	27.6	27.3	25.6	24.1	24.7	24.4	24.7
Average *B*-factor (protein)	55.6	64.3	93.0	92.9	76.2	42.6	46.0	36.1	35.3
Average *B*-factor (RNA)	72.2	87.4	114	91.4	111	75.6	76.5	65.7	48.1
R.m.s.d bond length (Å)	0.009	0.009	0.009	0.010	0.009	0.008	0.008	0.008	0.009
R.m.s.d bond angle (°)	1.13	1.22	1.25	1.29	1.22	1.10	1.12	1.10	1.12
Protein atoms^2^	3701	3701	3703	3703	3700	3693	3693	3690	7370
Nucleic acid atoms^2^	716	709	772	490	760	667	737	719	1964
Water molecules in cell	238	98	57	44	109	729	372	1246	408
Complexes per unit cell	2	2	2	2	2	4	4	4	4

1 Data in parentheses are for highest resolution shell.

2 Atoms per elongation complex prior to applying NCS constraints – see Figure 1 for NCS relationships used in refinement.

## Results

To crystallize elongation complexes with coxsackievirus B3 (CV), human rhinovirus 16 (RV), and poliovirus (PV) polymerases we sought to further explore the use of RNA-RNA stacking as a mediator of lattice contacts by systematically varying the length of the RNA and weakening the RNA-RNA contacts. These alterations to the RNA would change both the overall length between the predicted pairs of ECs in the lattice and the flexibility of the central region where the two RNAs interact by coaxial stacking. Changing the viral polymerase, on the other hand, is effectively another crystallization screening parameter that explores differences in the surface residues of the proteins to facilitate crystallization. Together, these two parameters allowed us to readily crystallize all three ECs tested. The resulting structures of these highly homologous polymerases sample a wide variety of crystallization conditions, allowing us to determine what parts of the EC structures are universally conserved and what parts may be due to crystal packing effects.

All ECs were formed using the previously described polymerase initiation protocol based on the addition of a (GA)_2–3_ repeat sequence onto a synthetic RNA primer strand in order to generate stably “locked” ECs that could be purified for crystallization [7]. To explore different lengths of upstream RNA duplexes we used different length primer-template pairs and locking sequences. All the RNAs shared a common downstream template strand sequence consisting of a 6-base pair hairpin with a GNRA-type tetra-loop (Figure 1). Stable complexes assembled using PV, CV, and RV polymerases could all be purified by high-resolution anion exchange chromatography. Elution was done with a linear gradient of monovalent salt and analysis of the absorbance chromatograms obtained at 260 and 280 nm and the final yield indicated that 50–70% of the input RNA was typically recovered in the form of stable purified ECs.

### Crystal Lattices

ECs were assembled on five different primer-template RNA sequences designed to systematically alter their potential for mediating crystallization contacts (Figure 1). Using the **r1** RNA used to crystallize and solve the initial PV polymerase EC structure (i.e. PV_r1), we were able to also crystallize the RV polymerase EC (RV_r1). The RV EC structure retained the coaxially stacked EC pairs seen with the original poliovirus structure, but note that the two RV EC pairs are in a very different spatial arrangement than previously observed. Next, we sought to slightly weaken the energetics of the coaxial stacking interactions that held two complexes together in the dumbbell geometry by replacing the 5**′** terminal GC dinucleotide overhang with a UG sequence. Replacing two G-C pairs with more flexible G-U pairs weakens, but does not eliminate, the potential for base-pairing interactions at the coaxial junction. Using this **r2** RNA, we were able to obtain crystals of the CV polymerase EC under two conditions but in the same crystal form, resulting in a 2.4 Å resolution structure (CV_r2) and a 2.2 Å resolution structure (CV_r2_Mg) that clearly showed a Mg^2+^ ion pre-bound to the coxsackievirus polymerase EC in the absence of an added NTP. Expanding on the **r2** construct, the length of the upstream RNA duplex between the two ECs was shortened from 26 to 24 (**r3**) and 22 (**r4**) base pairs, and both these RNAs resulted in poliovirus EC crystals that diffracted to 2.4 Å (PV_r3) and 2.8 Å (PV_r4) resolution. Last, we further extended the length of the RNA duplex to 30 base pairs with a longer (GA)_3_ locking sequence in the **r5** RNA and were able to obtain a third structure of the PV polymerase EC at 2.9 Å resolution (PV_r5). As further discussed below, in some cases NTP or deoxy-NTP soaks of these crystals resulted in catalysis and translocation, providing three additional structures of the PV and CV complexes.

**Figure 2:**
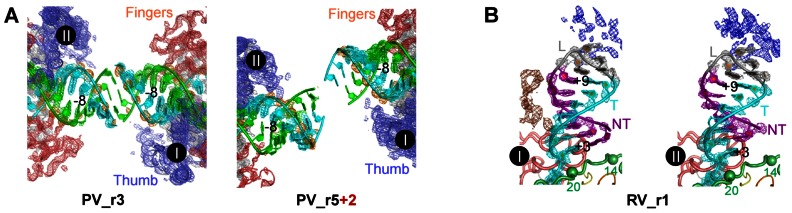
Electron density at RNA-mediated inter-complex crystal contacts. **A)** 3500 K composite simulated-annealing (SA) omit electron density maps (contoured at 1.5 σ) of the RNA-RNA junctions responsible for forming the dumbbell EC pairs. The left image is of the straightest junction (PV_r3) and the right image is of the most distorted junction (PV_r5+2). Note that despite the distortion and weak density around the junctions themselves, the upstream duplex largely maintains its A-form conformation as it exits the polymerase between the thumb and fingers domains. RNA strands and the associated density are color coded as in Figure 1 with phosphate density highlighted in orange. Polymerase molecules are shown in a three-color scheme with thumb, fingers, and palm domains in blue, red, and grey, respectively. **B)** Crystal packing contacts stabilizing the orientation of the downstream RNA stem-loop in the two different RV_r1 ECs. The 3500 K SA-omit electron density maps (1.5 σ) show electron density from two additional polymerase molecules (brown and blue) that make non-specific interactions with the tetra-loop and the downstream duplex. The RNA is colored as in Figure 1, the polymerase index (green) and pinky (pink) finger regions that form the cleft on which the downstream RNA duplex rides are shown as thick noodles, and the α-carbon atoms of the index finger residues 14 and 20 are shown for orientation.

**Figure 3:**
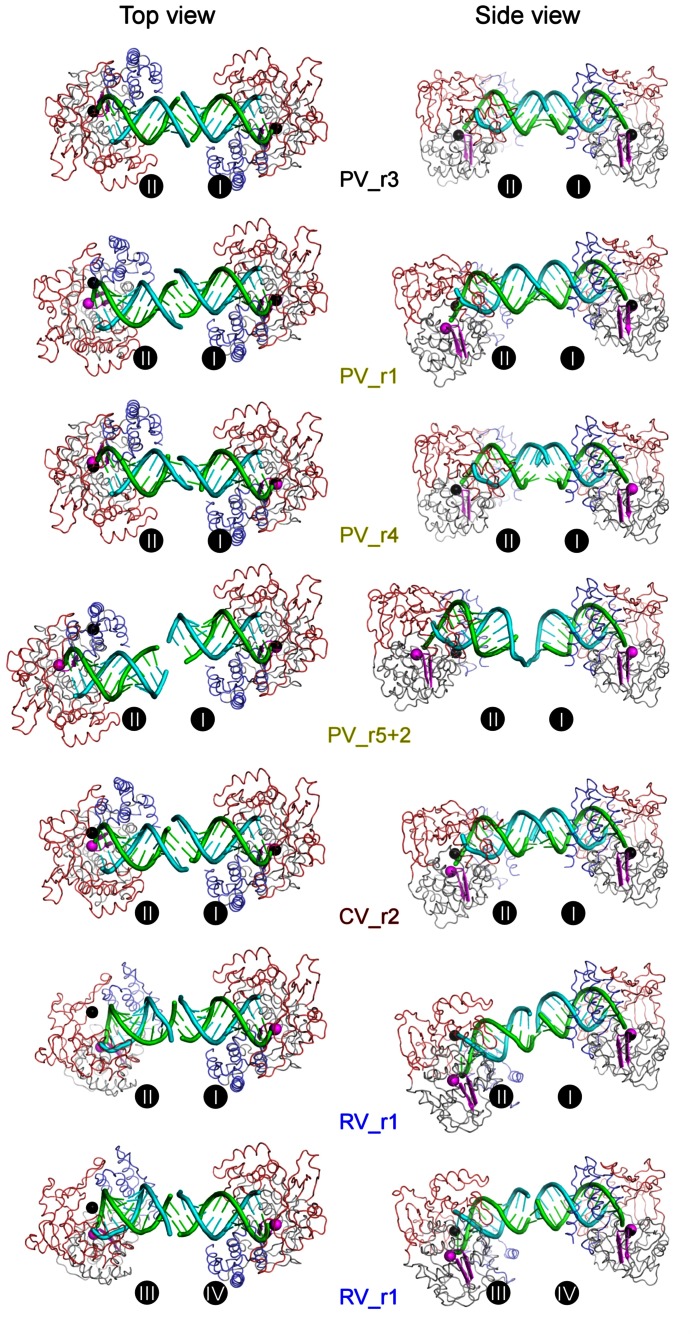
A comparison of inter-complex RNA-RNA junctions. Cartoon representations of seven EC pairs depicting the variety of inter-complex junctions observed in the crystals. The right side polymerase within each pair is always shown in the same orientation. The polymerases and RNA follow the color-coding scheme from Figure 1 with the YGDD residues of conserved motif C colored in magenta to help locate the polymerase active site. The PV_r3 structure (top) is used as a reference with its active site Asp328 Cα atoms shown as black spheres. These two black spheres are then overlaid onto the other complex pairs where the Cα atoms of the corresponding Asp residues are shown in magenta to highlight the differences in the placement of the non-superimposed left-side ECs. Non-crystallographic symmetry relationships show that all ECs except RV_r1 obey a pseudo two-fold symmetry within each dumbbell pair. See Figure 4 for a superpositioning of all the complexes.

All of the complexes crystallized with the anticipated coaxial stacking of the upstream RNA helices, demonstrating that the formation of the “dumbbell” orientation within EC pairs plays a critical role in establishing the crystal contacts. Despite this common lattice organization, the different dumbbell pairs and their RNA-RNA junctions displayed a multitude of geometries in the different crystal forms; for example, the junctions in the PV EC structures range from near perfect coaxial stacking to highly disordered structures that could not be fully modeled into the electron density maps (Figure 2A). A comparison of all the RNA-RNA junctions, shown in Figure 3, reveals geometries ranging from fairly straight and essentially canonical coaxial stacking (PV_r3, CV_r2) to slightly offset helical ends (PV_r1) to highly distorted geometries with frayed ends (PV_r5+2). As the junctions became increasingly non-canonical, the ends of the RNA helices exhibit weak electron density, suggesting the junction was more of a loose entanglement of RNA base and ribose groups, rather than a well defined set of interactions between two A-form helices. Importantly, these data show that the RNA-RNA junctions are not rigid anchor points, but rather joints with enough flexibility to allow for protein-protein contacts to play a key role in establishing the long-range lattice contacts needed for crystal growth.

The structures were all solved in the space group P1 and non-crystallographic symmetry (NCS) restraints were applied to related complexes during refinement (see Methods). This approach was build on our experience with the earlier PV EC structures where the unit cell dimensions were very close to C2 symmetry, but NTP soaking experiments revealed differences in the catalytic abilities of the different EC pairs due to differences in crystal packing environments [7].

### Downstream Template RNA Interactions

**Figure 4:**
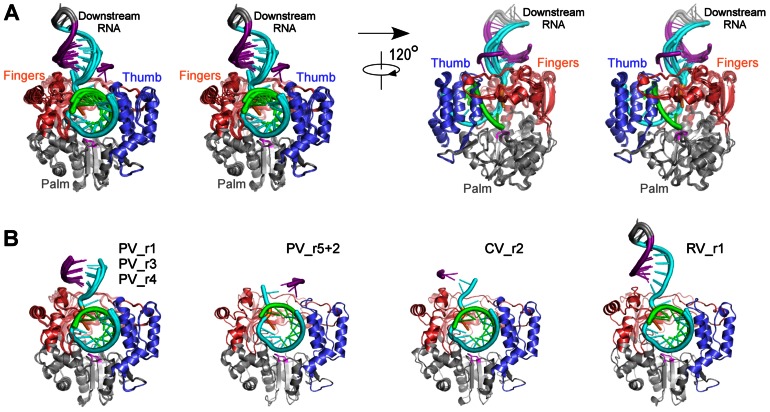
Comparison of EC structures and downstream RNA conformations. **A)** Stereo images of all eight picornaviral EC superimposed and shown in orientations along the upstream product duplex (left) and looking into the NTP entry channel (right). The RNA is color coded as in Figure 1 and polymerases follow the three-color coding scheme with the active site YGDD residues shown in magenta. Upstream product duplex RNA nucleotides that are completely outside of the front channel (position −9 and beyond) are omitted for clarity. Note that the position of the downstream RNA duplex, protruding from the fingers domain, is consistent in all complexes except for PV_r5+2 where this RNA is rotated by almost 180° and the 5**′** end is positioned above the thumb domain. **B)** ECs grouped according to virus species. From left to right: four PV complexes that are consistent in both polymerase and RNA conformation (two ECs from PV_r1 crystals); one PV complex that differs significantly from other PV complexes in downstream RNA conformation; one CV complex; two RV_r1 complexes that are differ slightly in downstream RNA conformation due to differences in crystal contacts (see Figure 2B).

The RV_r1 EC is the only structure where we observe significant contacts involving the downstream template duplex, but these appear to be non-specific interactions with other polymerase molecules in the lattice and not a biologically relevant interface (Figure 2B). The contact stabilizes the orientation of the RNA helix, resulting in strong electron density into which we could build the entire 6-base pair hairpin and tetra-loop sequence. In the remaining structures, as in our original PV_r1 poliovirus EC structure, we only observe 2–4 base pairs of the downstream helix with sufficient density to allow for model building (Figure 4). The density gets progressively worse with increasing distance from the polymerase and we interpret this to mean that the downstream duplex is tipping about an anchor point at the polymerase surface such that the more distal parts of the RNA swing through a greater arc and thus have weaker average electron density. The downstream RNA helix is typically oriented such that the non-template strand ends atop the fingers domain at a site near a channel that has been implicated in strand separation and single-stranded RNA template entry [28]. However, the 5**′** overhang strand is not long enough to form any well-defined interactions with the polymerase surface in any of the EC structures, suggesting that its orientation is to some extent influenced by crystal packing interactions. In fact, the PV_r5 structures shows the downstream template RNA helix rotated by ≈180° such that the 5**′** end of the non-template strand sits atop the fingers/thumb domain junction, but again there appear to be no well-defined interactions locking the RNA into this alternate orientation.

### Upstream RNA Interactions

**Figure 5:**
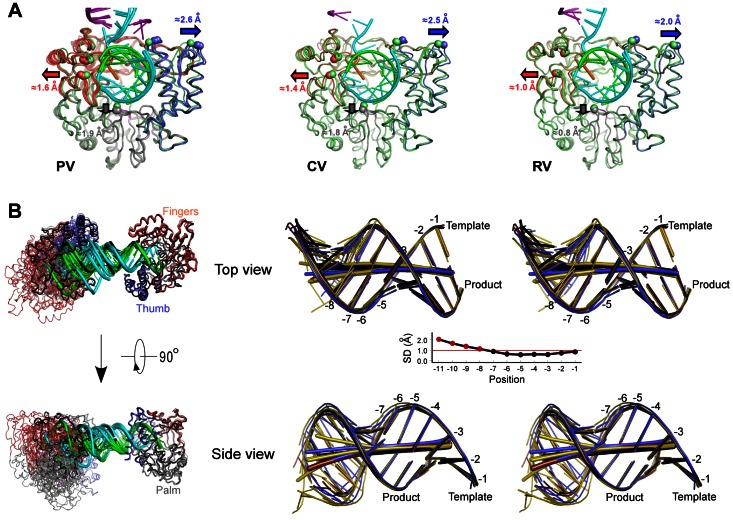
Polymerase front channel opening and conservation of RNA conformation. **A)** Opening of the polymerase front channel to accommodate the exiting RNA shown by the comparison of polymerase structures in their apo and elongation complex forms. All ECs follow the three-color coding scheme while the apo polymerases are shown in green. From left to right: all five PV ECs compared to one apo form (PDB code 1RA6); one CV EC compared to two apo forms (PDB codes 3DDK and 3DCU); two RV ECs compared to two apo forms (PDB code 1XR7 chains A & B). The major conformational differences are highlighted by blue, pink, and grey arrows indicating the movement within the thumb, fingers, and palm domains, respectively. The α-carbon atoms and displacement distances of PV polymerase residues 408 and 440 in the thumb, 102 and 133 in the pinky finger, and 211 in the palm, and their structural equivalents in CV and RV polymerases, are shown as spheres to further highlight the movements. **B)** Maximum likelihood superimposition of all eight distinct (i.e. not NCS-related) EC dumbbell pairs with the protein backbone Cα atoms of the right side polymerase being used for the alignment. The RNA from the PV_r3 EC pair that contains the most “straight” RNA-RNA junction is shown in black for reference, other complexes are colored as in Figure 2, and the downstream template RNA is omitted for clarity. Two views are shown to better depict the three dimensional variation of the partner complexes, with the “Top view” looking down into the active site and the “Side view” looking into the NTP entry channel. **C)** Stereo images of the upstream RNA duplex positions resulting from the protein-based structure alignment shown in panel B. The referencing PV_r3 duplex is in black, other PV duplexes are in yellow, CV duplex is in brown, and RV duplexes are in blue. The RNA helical axes as calculated by nucleic acids analysis program Curves+ are shown in thick sticks using the same coloring scheme. Consistent RNA conformations are observed from position −1 to position −8, with significant lateral variation of the base pairing starting around the −7/−8 base pair step. The middle panel shows the standard deviation of the RNA axis positions at each base step, mathematically suggesting that the upstream duplex conformation is only affected by the variation at the inter-complex junction beyond the −8 position. This corresponds to the point at which the RNA duplex exits from the polymerase and no longer contacts the thumb domain.

The ten total EC structures containing three different viral polymerases show a number of common protein-RNA interactions, indicating that these are highly conserved features of picornaviral RdRP complexes. All the complexes feature an upstream RNA duplex that is pinched between the pinky finger and thumb domains such that the RNA exit channel is opened by ≈4 Å as compared to the polymerase alone structures (Figure 5A). This tight grip on the exiting duplex is likely responsible for the observed high temporal stability and resulting high processivity of the picornaviral ECs [10], [29], [30]. The large number of EC structures we have now crystallized and solved using the purified stalled EC approach enables us to make direct comparisons among them and thus identify key regions of RNA contacts and RNA structure that are conserved among picornaviral polymerases. This analysis is aided by the fact that the picornaviral 3D^pol^ protein structures are by themselves highly homologous in the absence of RNA, allowing us to use the polymerase portion of the ECs to overlay the structures.

We used the program *Theseus* [31], [32] (www.theseus3d.org) to obtain maximum likelihood based superpositions of the protein portion of the different EC structures, and then we carried out a careful comparative analysis of the resulting RNA alignments. The advantage of the *Theseus* approach to structure alignments is that highly homologous regions of the protein structure are given more weight in the superpositioning while regions of the proteins that differ in structure (including single amino acid insertions and deletions) are down weighted, resulting in a tight alignment dominated by the most homologous elements of the polymerase structures. Figure 5B shows the superposition of all the dumbbell EC pairs where the right side polymerase was superimposed and the orientation of the other polymerase molecule within each EC pair is then dictated by the particular structure of the RNA and coaxial stacking junction. The resulting comparison reiterates the hereogeneity shown in Figure 3 and again demonstrates the wide range of relative EC orientations observed in the structures and further emphasizes that the RNA-RNA junctions are highly flexible and allow the proteins to form crystal lattices without constraining protein-protein interactions.

The analysis of the upstream RNA duplex structures shows that the first seven base pairs of the RNA helix are in essentially the same conformation in all seven crystal lattices that represent the EC structures of three different viral polymerases (Figure 5C). The RNA structures then begin to diverge at the −7/−8 base pair step based on the increase in the standard deviation of the helix axis position as calculated by the program Curves+ [33], and the helices exhibit clear and significant heterogeneity as one approaches their RNA-RNA junctions. These data indicate that the protein-RNA interactions and binding geometry are highly conserved as the RNA exits the EC along the thumb domain, where the 3D^pol^ α-helix composed of residues 406–420 lines the minor groove of the RNA duplex. It is only after this point that the structures diverge as a result of crystal packing contacts. Importantly, the great variation observed in the RNA-RNA junctions themselves does not affect the observed contacts between 3D^pol^ and the upstream duplex within the EC itself.

### In-Crystal Catalysis and Translocation

**Figure 6:**
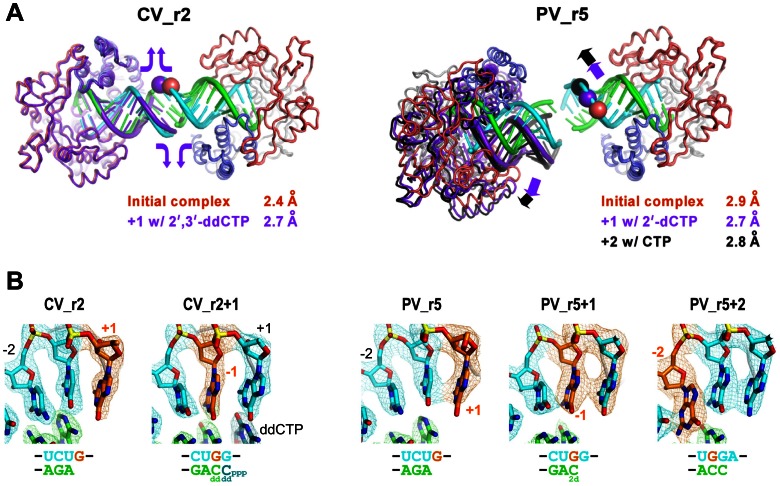
In-crystal translocation upon soaking with NTPs. **A)** Two different modes of accommodating in-crystal translocation via RNA-RNA junction flexibility. The right side polymerase chain has been superimposed and the multiple positions of the RNA and other ECs are shown in purple for +1 and black for +2 elongation states. The CV_r2 complexes undergo one translocation step with only minor changes in polymerase positioning because the junctions ends of the RNA unwind by one base per side to compensate, effectively keeping the upstream duplex length and crystal lattice constant. In contrast, the PV_r5 structure shows significant rotations of one EC relative to the other and repacking of the RNA-RNA junction as the crystal lattice is reshaped to accommodate the longer upstream duplex regions. **B)** Electron density maps of the template strand showing the movement of the guanosine originally found in the templating +1 position (orange) into the −1 and −2 positions on the product side of the active site. 3500 K composite SA-omit maps contoured at 1.5 σ are shown for the CV_r2 complexes where soaking with 2′,3′-ddCTP results in one incorporation and translocation event and the binding of a second ddCTP in the active site. 2F_o_–F_c_ maps contoured at 2.0 σ are shown for the PV_r5 structures where addition of 2′-dCTP results in one incorporation and translocation event while CTP addition results in two cycles of incorporation and translocation.

Nucleotide soaking experiments show that two of the lattices allow for catalysis and in-crystal translocation of the RNA. A key element of the resulting post-translocation structures is that the overall crystal lattice remains intact due to the plasticity of the RNA-RNA junctions (Figure 6). In the context of the dumbbell EC pairs, translocation should increase the inter-EC spacing by ≈2.7 Å and twist the two ECs relative to each other by ≈33°, respectively, per base and per elongating complex due to the A-form RNA helices linking them. Such movement would normally destroy the crystal lattice altogether, and we do in fact observe visible crystal cracking upon NTP soaks in some crystal forms. However, the PV_r5 and CV_r2 crystal forms continue to diffract and the resulting structures show they accommodate translocation in two different ways that further illustrate the utility and flexibility of the engineered coaxial stacking interactions as a crystallization tool (Figure 6).

Soaking the CV_r2 crystals with CTP results in cracking and a low resolution dataset (≈3.4 Å) that is not of sufficient quality to elucidate structural details, but soaking with 2′,3′-dideoxy-CTP (ddCTP) leads to a 2.7 Å resolution structure (CV_r2+1) reflecting one translocation event and a second ddCTP bound in the active site. This complex is stalled at the pre-catalysis state **2** of the catalytic cycle because the primer no longer has a reactive 3**′-**hydroxyl group following the first ddCTP incorporation event. The two polymerase molecules at opposite ends of the dumbbell remain essentially fixed and the translocation is accommodated by “peeling” nucleotides away from the end of the duplexes at the RNA-RNA junction so that they become disordered in solvent channels and are no longer resolved in the structure. There are minor perturbations at the end of the RNA helices as new non-canonical base stacking and hydrogen bonding interactions are formed by the nucleotides that have now been translocated into the junction regions.

The nearly opposite behavior is seen in the PV_r5 lattice, where one and two translocation steps in each EC leads to a systematic movement of one polymerase relative to the other that is accommodated by twisting and sliding movements at the RNA-RNA junction point. Soaking with 2′-deoxy-CTP (2′-dCTP) results in a single incorporation and translocation event and a state **1** structure without bound NTP, while addition of CTP results in two translocation steps utilizing both guanosines in the template strand. The original junction in the PV_r5 lattice is interesting because while there is density for the nucleic acid phosphate backbone, the bases themselves are only weakly ordered and appear to be loosely associated in multiple conformations. We thus consider this junction to be “sticky” without being highly structured, and it is this stickiness that presumably allows it to accommodate both translation and rotation of the polymerases into a new relative arrangement without destroying the crystal lattice altogether.

### Conservation of Active Site RNA Interactions

**Figure 7:**
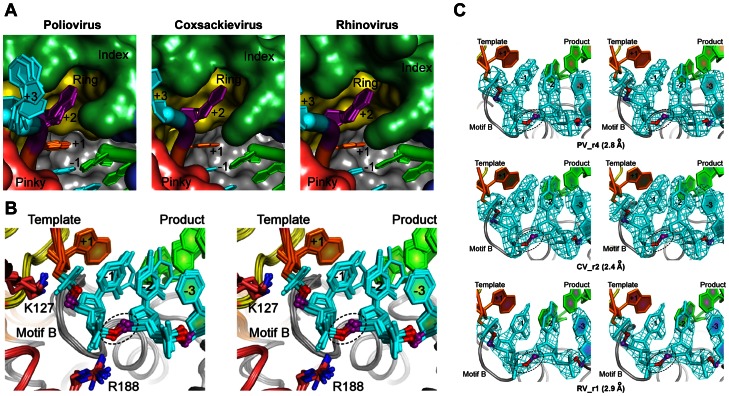
Conservation of RNA active site conformation. **A)** Top view looking at the downstream template RNAs from all ECs as it enters the active site. The +2 template strand nucleotide (purple) is consistently bound in a pocket formed by the polymerase index and ring fingers and the templating +1 nucleotide (orange) is always fully pre-positioned for base pairing with an incoming NTP in the active site. **B)** Stereo images of the RNA template strand conformation from positions −3 to +1 showing RdRP motif B positioned directly beneath the -1 position ribose of the template strand. The backbone of the -2 template strand nucleotide always has a different conformation than the neighboring A-form RNA helix nucleotides that is due to a 120° rotation about its ribose C5**′-**O5**′** bond (dashed circle). **C)** Stereo images of composite SA-omit electron density maps (3500 K, 1.5 σ) showing RNA density in the vicinity of the conserved non-standard backbone conformation of the −2 template strand nucleotide.

Within the active sites themselves, the templating +1 nucleotide is always found fully stacked on the upstream RNA duplex and prepositioned for hydrogen bonding interactions with the incoming NTP. The next templating nucleotide, i.e. the downstream +2 position, is found above the active site where its base moiety is bound in a pocket formed between Pro20 and Lys22 from the index finger (Figures 7A). At the product side of the active site we observe strong electron density for a conserved and non-canonical A-form backbone orientation of the phosphodiester linkage between the −1 and −2 nucleotides of the template strand (Figures 7B and 7C). This conformation is due to buried salt bridges between Lys127 and the −1 phosphate and between Arg188 and the −2 phosphate, and together these interactions hold the −1 ribose in tight contact with the conserved RdRP motif B (see [7] for distinction form motif B′) located at the junction of the palm and fingers domains. This RNA backbone conformation also appears to be important for properly positioning both the priming base pair and the templating nucleotide in the active site.

**Figure 8:**
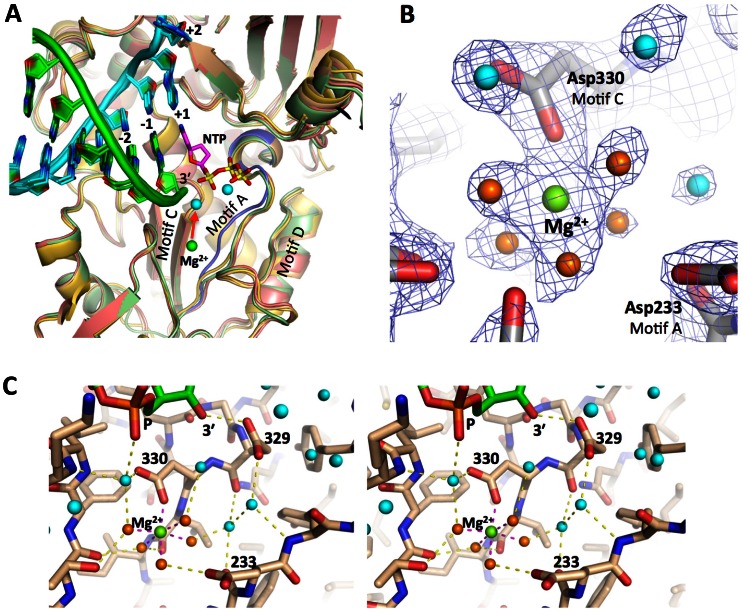
Motif A conformation and polymerase bound Mg^2+^ ion. **A)** Composite overview of the picornaviral polymerase active site based on a combination of superimposed structures to show the conformation of key conserve sequence motifs-A, -C, and -D involved in catalysis, a bound ddCTP (magenta) from the prior state **2** structure (PDB code 3OLB), and the positions of the catalytic magnesium ions before and during catalysis (green and cyan spheres). The structures presented in this paper (PV in red, CV in green, RV in gold) were all solved in the *open* conformation active site where motif A does not fully participate in the β-sheet with motif C, as shown by the comparison with the *closed* conformation of motif A (blue) seen in the prior state **4** structure (PDB code 3OL7). The CV_r2_Mg structure showed a magnesium ion (green sphere) pre-bound to the polymerase in a site 5.3 Å away from its position in the *closed* active site complex where it is joined by a second magnesium form the NTP-Mg complex (cyan spheres, from 3OL7). **B)** Electron density for the pre-bound magnesium ion coordinated by Asp330 and five water molecules (orange) in the CV_r2_Mg structure. Data is a 2F_o_-F_c_ electron density map contoured at 1.7 σ and Mg^2+^–O coordination distances are 2.0–2.2 Å. **C)** Stereo pair images of the Mg^2+^ coordination geometry and neighboring residues, including the RNA 3′ hydroxyl group, the active site Asp329 residue, and Asp233 from motif A that swings toward the active site to directly coordinate the magnesium in the *closed* conformation.

All of the complexes show the *open* conformation for the active site wherein the β-strands of motifs A and C are not hydrogen bonded together to fully form the RMM-fold that is commonly utilized by polymerase active sites (Figure 8A). In the CV_r2_Mg structure we observe clear density for a six-fold coordinated magnesium ion bound to the polymerase at a site about 5 Å away from the active site itself (Figure 8B). This is at a site that is commonly occupied by divalent metals in a number of other structures of the polymerase alone, and in several of the EC structures presented here the site is occupied by what is either a magnesium ion or a water molecule. These two atom types are difficult to distinguish based on electron density alone, but the clear six-fold coordination by closely spaced (<2.2 Å) water and side chain oxygen atoms seen in the CV_r5 structure definitively identify this as a magnesium binding site [34] (Figure 8C).

## Discussion

The initial structure of the poliovirus polymerase EC provided insights into the path of the template and product RNA strands as they threaded through the active site and showed that viral RdRPs use a unique palm-based structural change to close their active site for catalysis [7]. A comparison of available RdRP structures further showed that this palm-based mechanism is likely used by all the positive-strand RNA virus polymerases and we hypothesized it evolved because the fingers domains of these polymerases make a key structural contact with the top of the thumb domain that tethers the fingers, precluding swinging motions. In this work we extended on these findings by solving new structures of stalled elongation complexes with coxsackievirus and rhinovirus polymerases as well as obtaining three new crystals forms of the poliovirus polymerase complex. In combination with prior co-crystallization based structure of polymerase-RNA complexes from FMDV [35]–[37], norovirus [8], and hepatitis C virus [38], these structures reveal common protein-nucleic acid interaction sites, structural changes in the polymerases needed to accommodate the bound RNA, and conserved features of and interactions within the RdRP active sites.

Using a small library of different length RNA constructs, we were able to crystallize all three polymerases tested in multiple crystal forms. In all cases, the crystal lattices were dominated by the RNA-RNA interactions that resulted in pairs of ECs arranged into dumbbell type structures. Overall, the RNA-RNA packing interfaces showed structural heterogeneity, divergence from a canonical A-form helix, and weakened electron density as one approaches their junction regions. Thus, the coaxial stacking interactions exhibit significant plasticity, providing for a loose association of ECs that is critical for nucleating crystallization while at the same time providing enough flexibility for long-range crystal lattice formation via additional protein-protein contacts. This flexibility is further emphasized by the finding that two of the lattices allow for in-crystal RNA translocation in NTP soaking experiments, resulting in new structures with the potential to highlight intermediates in the polymerase elongation cycle.

The resulting set of ten different EC structures allows us to make direct comparisons of the different enzymes and further distinguish between conserved protein-RNA interactions found within all the picornaviral ECs and other conformations that are likely the result of crystal packing effects. This comparison can also be expanded to include similar structures from related viruses, allowing us to further highlight commonalities of viral polymerase-RNA complexes. The comparison shows that the RdRPs tightly associate with 7–8 base pairs of the product RNA duplex in an A-form helix that is held in place by an upstream clamp between the pinky finger and an α-helix from the thumb domain that packs into the minor groove. All three picornaviral polymerases exhibit a ≈4 Å opening of this channel in their EC structures as compared to their structures without RNA, indicating that the clamp structure holding the RNA product is a common feature of these polymerases (Figure 5A). On the downstream template side there is a conserved binding pocket for the +2 nucleotide and this anchoring interaction probably acts in concert with the upstream clamp to establish the high temporal stability of the ECs. In contrast, there are no indications of conserved protein-RNA interactions further downstream of the +2 position; all the RNAs tested included a downstream duplex, but only in the RV_r1 structure did we observe sufficient electron density to model the full downstream RNA hairpin due to the fact that the RV_r1 helix is held in place via non-specific interactions with other polymerases in the crystal lattice (Figures 1 and 2B). We can only model the first two to four downstream base pairs in the other EC structures, suggesting that the downstream helix is not tightly held in place and instead pivots about point where the template strand dips down into the active site. Consistent with this, the set of PV_r5 structures show the downstream helix rotated by ≈180° such that the 5**′** end of the helix is closer to the thumb domain than the fingers domain (Figure 4B), but again there are no specific polymerase-RNA interactions that lock the nucleic acid into this particular orientation.

The EC structures also allow for extensive comparisons of the RdRP active sites and the geometry of the RNA as it passes through the polymerase. All the structures were captured in state **1** of the previously described [7] catalytic cycle with a fully pre-positioned templating NTP sitting above the active site poised for hydrogen bonding interactions with the cognate incoming NTP. As was observed in the initial poliovirus EC structure, all the structures have an *open* conformation active site where motif A is not fully participating in the core RRM-fold of the palm domain and its aspartic acid Asp233 residue that is responsible for coordinating both Mg^2+^ ions during catalysis is pointed away from the catalytic center. In the CV_r2_Mg structure we see clear density for a bound magnesium ion that is coordinated by Asp330 in motif C and five water molecules in a six-fold coordination geometry, with Asp233 from motif A being involved in the network (Figure 8C). This magnesium ion likely represents the polymerase bound Metal A ion that is moved into the active site to facilitate catalysis in conjunction with the Metal B magnesium that is brought in with the NTP [39], and it is bound in site where metals have been observed in several other viral polymerase structures even in the absence of RNA and NTPs [40]. The positioning of Arg174 above the triphosphate region of the NTP binding site is also conserved among all the structures and this hydrogen bonding of Arg174 to the NTP and pyrophosphate leaving group appears to facilitate catalysis based on quantum mechanical calculations [16]. All the structures also show an A-form conformation of the upstream RNA duplex whose minor groove is lined by an α-helix from the thumb domain. On the template strand side of the active site, the ribose groups of both the +1 (i.e. templating base) and −1 nucleotides are held in close contact with the conserved polymerase motif B located at the base of the fingers domain. This tight interaction is primarily due to ionic contacts between the +1 and -1 phosphate groups and Lys127 and Arg188 (Figure 7B), two residues that are essential for virus replication [28], as well as direct and water-mediated hydrogen bonds between motif B and the ribose backbone.

The core RNA sequence used to assemble the stable ECs contains a pair of templating guanosines such that adding CTP can potentially result in elongation of the primer by at most two bases. In our prior work on the poliovirus polymerase EC structure the CTP crystal soaking experiment resulted in a single round of catalysis but no translocation of the RNA took place and the polymerase was trapped with its active site in the closed conformation. We hypothesized that the lack of translocation was due to crystal lattice constraints, and consistent with this we now observe translocation in two of the new lattices that are formed using weaker G-U base pairs in the coaxial stacking junction (Figure 6). A CTP soak of the poliovirus 3D^pol^ PV_r5 crystal resulted in two nucleotide incorporation events and two translocation steps, giving a final state **1** structure that is essentially identical to the starting point, only with sequence variation in the RNA strands. The RNA-mediated lattice contacts of this crystal form allowed for significant twisting of one EC relative to the other within the dumbbell pair, but the structure within each EC was essentially unchanged. In contrast, the coxsackievirus 3D^pol^ CV_r2 lattice accommodated one translocation event upon 2′,3′-ddCTP addition by keeping the two ECs within a dumbbell pair essentially fixed while unraveling the ends of the two RNA duplexes at the junction point. These structures are both static snapshots of a post-translocation state that is essentially in the same conformation as the starting structure and therefore do not provide any insights into the details of how translocation is achieved in these viral polymerases. However, the structures do suggest that a straightforward NTP soaking experiment should always be employed as a simple method for sampling additional conformation states that may yield structures for such translocation intermediates in the future.

In summary, we have used a small set of five different RNA constructs to solve nine different picornaviral polymerase elongation complex structures. These structure show conservation of active site interactions between the polymerase and the RNA, such as the pre-positioned templating +1 base, the index finger binding pocked for the +2 nucleotide, the unique conformation of the template strand −1 to −2 phosphate linkage, a lack of significant interactions with the downstream template, and a consistent ≈4 Å opening of the RNA exit channel as compared to the polymerases in the absence of RNA. The structures presented here also demonstrate that engineering flexibly stacked RNA helices is a powerful experimental tool for the crystallization of polymerase ECs. The coaxial stack RNA-RNA interactions provide for a key association of complexes into dumbbell type structures that likely promote lattice nucleation, but they retain enough flexibility to allow a crystal lattice to grow via protein-protein contacts. The assembly and purification of the stable ECs themselves is straightforward for the primer dependent picornaviral polymerases, and the recent development of a method for generating similar complexes with the *de novo* initiating hepatitis C virus polymerase [41] sets the stage for similar studies of flaviviral polymerases. Such structures would complement those obtained by soaking smaller RNAs into pre-formed crystals of polymerases lacking the autoinhibitory β-loop required for *de novo* initiation [38], [42]. Finally, the downstream RNA template strand sequence can be designed to allow for controlled in-crystal catalysis, laying the groundwork for further studies to address the molecular basis of fidelity and translocation in these pathogen polymerases.
